# Stent graft treatment for ruptured pseudoaneurysms of the splanchnic arteries after pancreaticoduodenectomy: a case report

**DOI:** 10.1186/s40792-020-00887-w

**Published:** 2020-06-01

**Authors:** Masaru Nemoto, Ken Koyama, Midori Enokido, Shohei Kudo, Fuyo Yoshimi

**Affiliations:** 1 Department of Surgery, Ibaraki Prefectural Central Hospital & Cancer Center, 6528 Koibuchi, Kasama, Ibaraki, 309-1793 Japan; 2 Department of Diagnostic and interventional radiology, Ibaraki Prefectural Central Hospital & Cancer Center, 6528 Koibuchi, Kasama, Ibaraki, 309-1793 Japan; 3 Department of Hepato-Biliary-Pancreatic Surgery, University of Tokyo, 7 Chome-3-1 Hongo, Bunkyo City, Tokyo, 113-8654 Japan

**Keywords:** Stent graft, Pancreaticoduodenectomy, Pseudoaneurysm

## Abstract

**Background:**

Hemorrhage due to a ruptured splanchnic pseudoaneurysm followed by the formation of a postoperative pancreatic fistula is the most severe complication of a pancreatectomy, sometimes leading to a fatal outcome. Stent graft placement to control the hemorrhage due to the pseudoaneurysm is a validated treatment option, but once the stent graft is infected, infection control is complicated. We report a case of a ruptured pseudoaneurysm of the splanchnic artery after pancreaticoduodenectomy to evaluate the stent graft treatment.

**Case presentation:**

A 77-year-old man underwent pylorus-preserving pancreaticoduodenectomy for suspected distal bile duct cancer. Hemorrhage from a pseudoaneurysm of the common hepatic artery due to the formation of the pancreatic fistula was detected on postoperative day 9, and a stent graft was successfully placed with the preservation of hepatic arterial blood flow. On postoperative day 12, new-onset hemorrhage from a pseudoaneurysm of the right hepatic artery developed, and a stent graft was similarly placed, but immediately occluded. Refractory pancreatic and biliary fistulas developed and required continuous drainage. On postoperative day 85, computed tomography revealed the presence of air within the latter stent graft, which indicated infection of the stent graft. The patient died due to sepsis caused by the graft infection.

**Conclusion:**

Stent graft placement for the treatment of hemorrhage of a pseudoaneurysm secondary to a postoperative pancreatic fistula, following pancreaticoduodenectomy, is an effective treatment option as it achieves immediate hemostasis and maintains end-organ perfusion. However, stent graft infection is the most detrimental complication.

## Background

Pancreaticoduodenectomy (PD) is a standard surgical procedure for the treatment of pancreatic head and distal bile duct disease. The mortality rate due to a PD has decreased to less than 5%, but the morbidity rate remains high, ranging between 30 and 50% [1]. Hemorrhage and postoperative pancreatic fistula (POPF) formation are the most severe complications following PD, sometimes leading to a fatal outcome. Delayed hemorrhage of ruptured splanchnic pseudoaneurysms caused by a POPF, which mainly occurs in the common hepatic artery (CHA) and the stump of the gastroduodenal artery (GDA), is a life-threatening condition with a high mortality rate and requires immediate intervention [2]. Surgical hemostasis and transcatheter arterial embolization (TAE) are the treatment options for the hemorrhage of pseudoaneurysms. Alternatively, treatment using stent graft placement has been sporadically reported and results in excellent clinical outcomes with the preservation of end-organ arterial blood flow [3]. Herein we report a case of ruptured splanchnic pseudoaneurysms secondary to a POPF following PD, which was treated with the placement of a stent graft to obtain primary hemostasis, but the stent graft was infected and lead to a fatal outcome.

## Case presentation

**Fig. 1:**
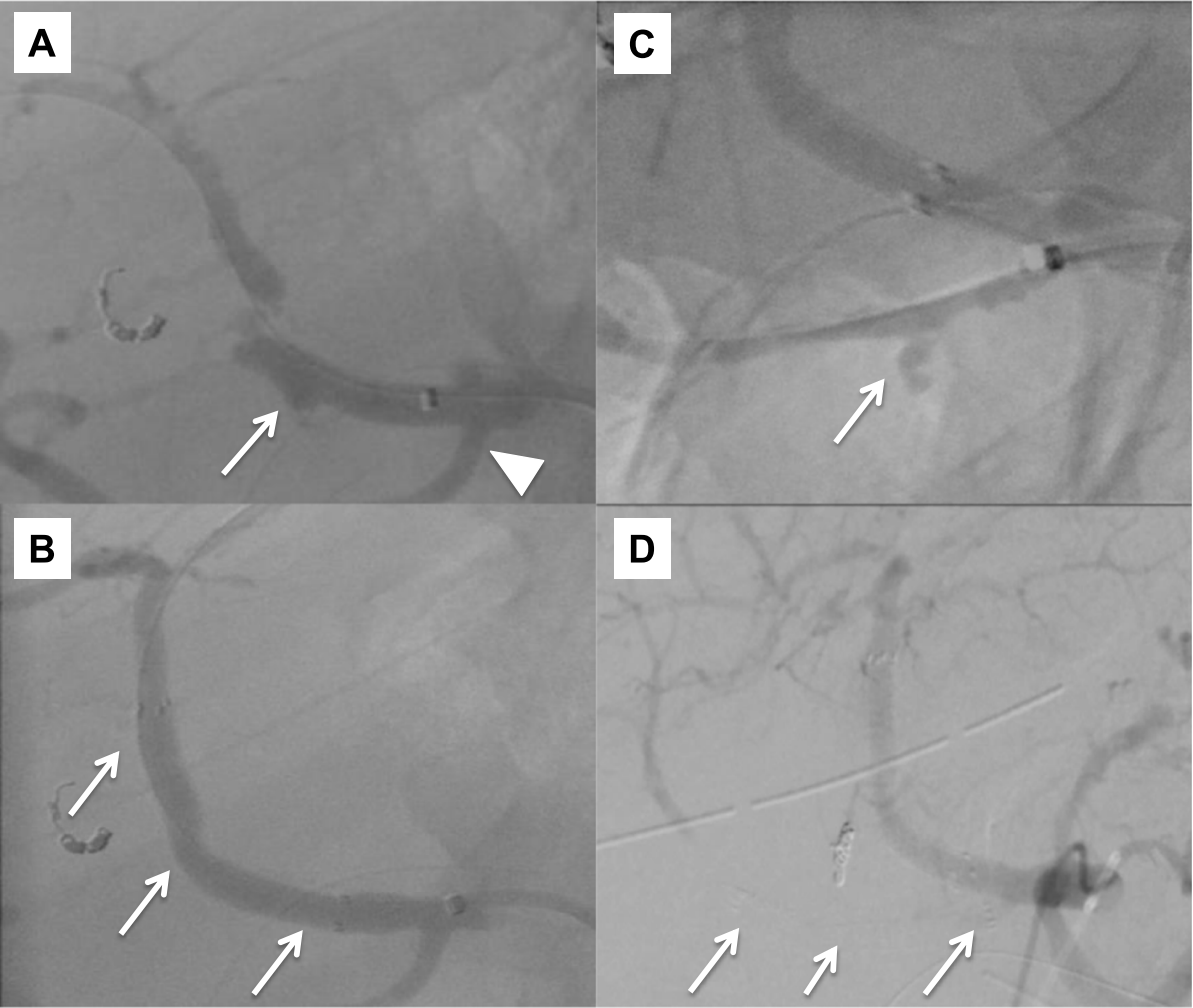
Pre- and postoperative angiogram. **a** Angiogram reveals contrast extravasation from the pseudoaneurysm at the CHA (arrow). The RHA is branched independently from the CHA (arrowhead). **b** A stent graft, 6 mm in diameter and 5 cm in length, is placed from the CHA and distal part of the replaced RHA to the LHA (arrows). **c** Angiogram shows contrast extravasation from the RHA (arrow). **d** A stent graft, 5 mm in diameter and 5 cm in length, was placed in the RHA, but occluded soon after (arrows)

**Fig. 2:**
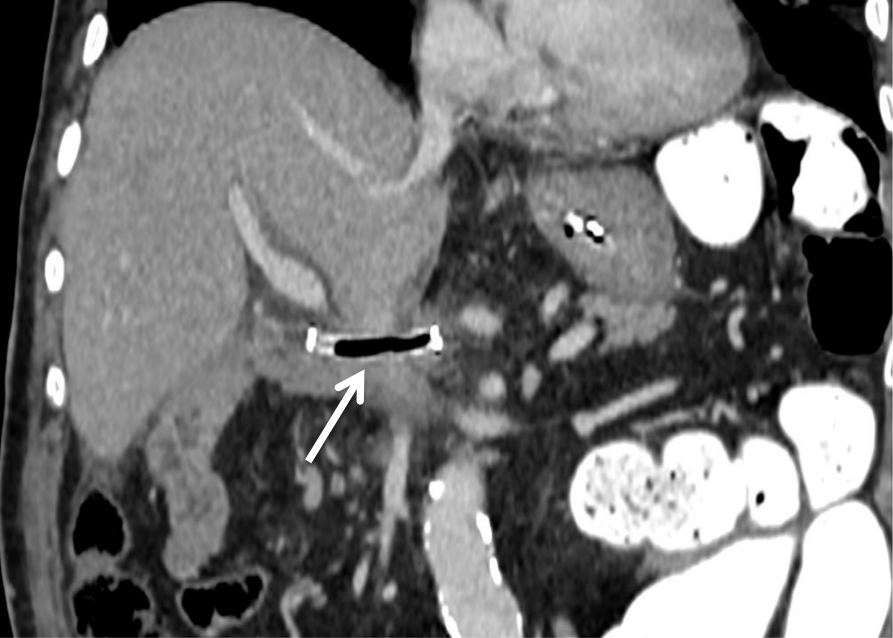
Enhanced CT on POD 85. CT reveals the presence of air within the occluded stent graft, which indicates stent graft infection (arrow)

A 77-year-old man, with a background of hypertension and diabetes mellitus, underwent pylorus-preserving PD for suspected distal bile duct cancer. He developed a pancreatic anastomotic leakage on postoperative day (POD) 6, which was draining from a drain placed at the pancreaticojejunal anastomosis during surgery. Sentinel bleeding from the drain was noted on POD 8, and an immediate contrast-enhanced computed tomography (CT) scan revealed no obvious bleeding points. However, a massive hemorrhage from the drain suddenly developed on POD 9, and emergency angiography detected a pseudoaneurysm at the CHA, which had caused the bleeding (Fig. 1a). The right hepatic artery (RHA) independently branched from the origin of the CHA. Based on the general condition of the patient and the likelihood of postoperative adhesions, the placement of a stent graft was the treatment of choice. A 6-F sheath was passed through the celiac trunk via the femoral artery. A 6 mm × 5 cm Viabahn stent (W.L. Gore, Flagstaff, Ariz) was placed from the CHA and the distal part of the replaced RHA to the left hepatic artery (LHA) with heparinization (Fig. 1b). Angiography following stent graft placement revealed the absence of a pseudoaneurysm and preserved hepatic arterial blood flow. His vital signs were stable after the intervention, and antiplatelet therapy was initiated using aspirin 100 mg/day. However, re-bleeding from the drain appeared on POD 12. Angiography was immediately performed, and contrast extravasation from an aneurysm at the RHA was detected (Fig. 1c). A 5 mm × 5 cm Viabahn stent was placed into the RHA with heparinization. Subsequent angiography showed the occlusion of the latter Viabahn stent, while the patency of the former Viabahn stent was maintained (Fig. 1d). Due to sufficient hepatic arterial blood flow through the RHA, by retrograde flow via the LHA, no additional intervention for the stent graft occlusion was performed, and the patient was carefully observed. A biliary fistula appeared on POD 15, which might be caused by the occlusion of RHA and compression of massive hematoma around the biliary anastomosis. An abdominal abscess around the biliary anastomosis was detected, and percutaneous CT-guided drainage was performed on POD 28, and antibiotic therapy based on a bacterial culture was initiated. These pancreatic and biliary fistulas were considered to be a refractory complication. On POD 85, the patient’s general condition worsened, and the CT scan revealed the presence of air within the latter occluded stent graft, which indicated that the stent graft had become infected (Fig. 2). *Escherichia coli* was confirmed by blood culture, and the patient died on POD 91 due to sepsis caused by the graft infection. This study was approved by the ethics committee of our institution, and informed consent was obtained from the patient’s next-of-kin.

## Discussion

Pseudoaneurysms are believed to be caused by anatomical leakages, mainly due to pancreatic fistulas or injury of the arterial wall during operative dissection and lymphadenectomy [4]. It is crucial to detect and initiate interventions in cases of hemorrhaging due to pseudoaneurysms in order to improve outcomes. In this case, we presumed that the local infection secondary to the POPF had caused the pseudoaneurysms in the CHA and RHA to develop.

Despite the identification of the sentinel bleeding and having performed the CT scan in our case, the pseudoaneurysms were not clearly detected, and angiography was canceled. Sentinel bleeding is considered a sign of pseudoaneurysm bleeding prior to massive hemorrhage and is often observed in 45–78% of patients who have undergone PD [5]. Angiography has been recommended if this critical sign is identified [6].

Surgical re-exploration is the conventional management of hemorrhage due to a pseudoaneurysm. However, the surgery is difficult to perform due to tissue fragility and postoperative adhesions, which results in a significant risk of mortality and morbidity. The operative mortality rate has been reported as 43–50%. Therefore, surgical re-exploration is recommended when an endovascular approach is difficult [7]. Endovascular treatments, including TAE and stent graft placement, have been accepted as alternative treatment options for post-pancreatectomy hemorrhage. The advantages of endovascular treatments are the ability to perform these treatments percutaneously and immediately without the need for general anesthesia. TAE has been performed with high success and a lower mortality and morbidity rate when compared to open surgery [8]. However, liver abscess formation and liver dysfunction due to end-organ ischemia and re-bleeding may occur, resulting in an increasing rate of mortality [8].

To avoid these complications, stent graft placement is preferred over TAE. Stent graft placement provides excellent flexibility, enables precise deployment of the stent, and maintains end-organ arterial blood flow. Several reports on stent graft placement for the treatment of pseudoaneurysms after PD have recently been published [9, 10]. Asai et al. reviewed 34 cases of stent graft placement for visceral delayed arterial hemorrhage after pancreatic-head resection, and the hemorrhage site was found to be the hepatic artery in 27 patients, the superior mesenteric artery (SMA) in 5 patients, and the splenic artery (SA) in 2 patients [11]. The technical success rate was 100%. The complications following stent graft placement included the development of a liver abscess (2.9%), re-bleeding (11.8%), and stent occlusion (5.9%). It has been recommended that double antiplatelet treatment (DAPT) be administered after stent graft placement in the visceral artery, for the prevention of stent graft occlusion. However, when hemorrhaging occurs, it is difficult to initiate the DAPT, and single antiplatelet treatment might be preferred. The morbidity associated with stent graft placement has been considered the lowest when compared to that in TAE and re-laparotomy. However, stent graft infection is the most detrimental complication [8]. Four cases with the development of sepsis following stent graft placement for visceral hemorrhage after PD have been reported. Only 1 case of stent graft infection following placement for a pseudoaneurysm of the SMA has been reported [3]. In this case, air was present in the occluded stent graft due to abscess formation around the SMA with an unhealed POPF 4 months after stent graft placement. The patient died due to sepsis 5 months after that. Three cases have been reported in which 2 patients have died due to intra-abdominal sepsis, and 1 patient had died due to an unknown cause (Table 1) [12–14]. It is presumed that the occlusion of the stent graft might be a risk factor for the development of an infection as the thrombus within the stent graft might act as a medium for bacterial growth. Uncontrolled local infection with a POPF or a biliary fistula is another risk factor for stent graft infection. The risk of infection is considered similar in TAEs and stent grafts [12]. In our case, the placement of the stent graft was selected based on the general condition of the patient and the anatomical location of the pseudoaneurysm. Although percutaneous drainage of the POPF and the biliary fistula was performed in order to control local infection, the contact of abscess formed by the refractory fistula development to the occluded stent graft could have caused stent graft infection. Limitations of this study include the retrospective study design and small sample size.

**Table 1:** Cases of stent graft infection or sepsis following stent graft placement for visceral hemorrhage after pancreaticoduodenectomy

Author	Year	Age	Sex	Bleeding point	Stent type	Outcome	Cause
Stoupis et al. [12]	2006	60	Male	CHA	Jostent graft	Died after 10 days	Sepsis (intra-abdominal sepsis)
Heiss et al. [13]	2007	72	Male	SA	Advanta	Died after 3 months	Sepsis (unknown)
Suzuki et al. [3]	2009	70	Female	SMA	Passager	Died after 5 months	Stent graft infection and sepsis
Wang et al. [14]	2010	53	Male	CHA	Jostent graft	Died after 12 days	Sepsis (intra-abdominal sepsis)
Present case	2019	77	Male	CHA, RHA	Viabahn	Died after 91 days	Stent graft infection and sepsis

*CHA* common hepatic artery, *SA* splenic artery, *SMA* superior mesenteric artery, *RHA* right hepatic artery

## Conclusions

Stent graft placement for the treatment of hemorrhage of a pseudoaneurysm secondary to a POPF after PD is an effective treatment option. It immediately achieves hemostasis and maintains end-organ perfusion better than that in TAE and surgical re-exploration. If local infection around the pseudoaneurysm is uncontrolled, and the stent graft is occluded, a lack of immediate infection control may lead to a fatal outcome.

## Data Availability

All datasets supporting the conclusions of this article are included in this published article.

## Abbreviations

PD: Pancreaticoduodenectomy
POPF: Postoperative pancreatic fistula
TAE: Transcatheter arterial embolization
CHA: Common hepatic artery
GDA: Gastroduodenal artery
RHA: Right hepatic artery
LHA: Left hepatic artery
POD: Postoperative day
SMA: Superior mesenteric artery
DAPT: Double antiplatelet treatment
SA: Splenic artery
CT: Computed tomography
